# Readiness to change is a predictor of reduced substance use involvement: findings from a randomized controlled trial of patients attending South African emergency departments

**DOI:** 10.1186/s12888-016-0742-8

**Published:** 2016-02-20

**Authors:** Bronwyn Myers, Claire van der Westhuizen, Tracey Naledi, Dan J. Stein, Katherine Sorsdahl

**Affiliations:** Alcohol, Tobacco and Other Drug Research Unit, South African Medical Research Council, Francie Van Zyl Drive, PO Box 19070, Tygerberg, 7505 Parow South Africa; Department of Psychiatry and Mental Health, University of Cape Town, J block, Groote Schuur Hospital, Anzio Road, Observatory, 7935 South Africa; Alan J Flisher Centre for Public Mental Health, University of Cape Town, 46 Sawkins Road, Rondebosch, 7700 South Africa; Western Cape Department of Health, 8 Riebeeck Street, Cape Town, 8001 South Africa; Anxiety and Stress Disorders Unit, South African Medical Research Council, Francie Van Zyl Drive, Parow, 7505 South Africa

**Keywords:** Readiness to change, Substance use, Motivation, Brief intervention, Emergency departments, South Africa

## Abstract

**Background:**

This study examines whether readiness to change is a predictor of substance use outcomes and explores factors associated with RTC substance use among patients at South African emergency departments.

**Methods:**

We use data from participants enrolled into a randomized controlled trial of a brief substance use intervention conducted in three emergency departments in Cape Town, South Africa.

**Results:**

In adjusted analyses, the SOCRATES “Recognition” (*B* = 11.6; 95 % CI = 6.2–17.0) and “Taking Steps” score (*B* = -9.5; 95 % CI = -15.5- -3.5) as well as alcohol problems (*B* = 4.4; 95 % CI = 0.9–7.9) predicted change in substance use involvement at 3 month follow-up. Severity of depression (*B* = 0.2; 95 % CI = 0.1–0.3), methamphetamine use (*B* = 3.4; 95 % CI = 0.5- 6.3) and substance-related injury (*B* = 1.9; 95 % CI = 0.6–3.2) were associated with greater recognition of the need for change. Depression (*B* = 0.1; 95 % CI = 0.04 -0.1) and methamphetamine use (*B* = 2.3; 95 % CI = 0.1 -4.2) were also associated with more ambivalence about whether to change. Participants who presented with an injury that was preceded by substance use were less likely to be taking steps to reduce their substance use compared to individuals who did not (*B* = -1.7; 95 % CI = -5.0- -0.6).

**Conclusion:**

Findings suggest that brief interventions for this population should include a strong focus on building readiness to change substance use through motivational enhancement strategies. Findings also suggest that providing additional support to individuals with depression may enhance intervention outcomes.

**Trial registration:**

This trial registered with the Pan African Clinical Trial Registry (PACTR201308000591418) on 14/07/2013.

## Background

The prevalence of substance use disorders is high in South Africa where an estimated 13 % of the adult population meet DSM-IV criteria for a lifetime diagnosis of a substance use disorder [1] and rates of heavy episodic drinking are among the highest in the world [2]. Untreated substance use disorders impact negatively on public health in South Africa through their association with risk for HIV and other infectious diseases [3], non-communicable diseases [4], and interpersonal violence and injury [5] - the main contributors to morbidity and mortality in the country [6]. For example, data indicate that between 33 and 79 % of patients presenting to South African emergency departments (EDs) have substance use-related injuries [7–9]. Although many of these patients will have recurring substance-related injuries [10], most have never sought formal substance abuse treatment [11]. Screening patients in EDs for substance use-related problems, and providing brief interventions (BIs) to at-risk patients may reduce the likelihood of further substance-related injuries [12].

Systematic reviews have generally found BIs delivered in health care settings to have efficacy for reducing alcohol consumption [13–16], although there is limited evidence of efficacy for heavy dependent drinkers and young people [15, 17, 18]. In contrast, there is currently insufficient evidence to draw conclusions about the effectiveness of BIs for reducing illicit drug use among non-treatment-seeking populations [19]. Recent trials conducted predominantly in the U.S. found that BIs had no significant effect on illicit drug use [20–23], although one study [24] found that individuals with more severe drug use problems had improved utilization of substance abuse treatment and lower utilization of emergency services subsequent to receiving a BI. However, all of these studies included participants with a variety of substance use problems. Although these studies did not explore whether type of drug was associated with response to the BI, it is possible that this could have accounted for these equivocal findings especially given evidence of a relationship between drug type and substance abuse treatment outcomes [25]. This variability in BI outcomes highlights the importance of understanding who is most likely to be responsive to BIs delivered in health care settings. This would allow for the design of more effective and efficient BIs by allowing these interventions to be optimally allocated to patients who are most likely to benefit, and by identifying ways to improve efficacy. Ensuring that interventions are effective and efficient are important goals for health care services in low-and-middle-income countries (LMIC) like South Africa where there are few resources to deliver substance use screening and BIs [26].

Motivation, or readiness, to change is thought to be a key facilitator of substance-related behaviour change. Several studies have demonstrated that readiness to change (RTC) is a predictor of response to BIs, with greater RTC associated with better outcomes [27–29]. However, these studies have several limitations. First, most of these studies have been conducted in high income countries and it is unclear whether these findings can be extrapolated to LMICs such as South Africa where patterns of substance use are substantively different to those typically seen in high income countries. For example, LMICs in Africa have significantly lower prevalence rates for cannabis and opioid dependence than high income countries [30, 31]. Additionally, in contrast to high income countries where frequent light drinking is typical, infrequent but heavy episodic drinking is the normative pattern in many African countries [2] and this may influence responses to measures of RTC [32]. Second, apart from a few studies [33–36], knowledge of how RTC informs response to BIs within the ED population is limited. Given the significant burden on the health care system posed by ED patients with substance-related problems [12], there is a need to better understand this population’s response to BIs for substance use.

Finally, previous studies have not examined factors potentially associated with RTC among substance-using patients presenting at EDs. Studies in other populations have shown that greater substance use problem severity [37–39] and negative physical consequences including injury [36, 37, 40] are associated with enhanced RTC. Mixed findings have been found for the association between socio-demographic factors (such as age, race and gender), and RTC substance use [29, 37]. While mental health problems such as depression are strongly associated with substance use problems [41] and several studies have shown that depression is associated with greater RTC substance use [42–44], others have found no significant association [41, 45, 46]. While methodological differences in the choice of RTC measures and depression screeners may account for these equivocal findings, the relationship between depression and RTC substance use, particular in ED populations, requires further study.

The current study seeks to expand upon previous studies by examining (1) whether RTC is a predictor of substance use outcomes among patients at South African emergency departments (ED) who received a BI to reduce substance use involvement and (2) by exploring socio-demographic and health-related factors associated with RTC substance use in this population. Reported here is a secondary data analysis of a randomised controlled trial of a screening, BI and referral to treatment (SBIRT) programme among ED patients at moderate and severe risk for substance-related health problems in Cape Town, South Africa [47].

## Methods

### Study design

In this study, 2736 adult patients presenting to three EDs in Cape Town, South Africa were screened for study eligibility between March 2012 and November 2012. Patients were screened at various times and during at least one 12-h night shift on the weekend to reflect the busiest periods of the EDs. Patients were eligible for study inclusion if they were ≥ 18 years of age and screened at moderate to high risk for substance use problems on the Alcohol, Smoking and Substance Involvement Screening Test [48]. Patients were not eligible if they had a severely altered mental status, were physically incapable of participating due to severe illness, or were unable to provide contact information.

Eligible patients (*n* = 443) were asked to consent to participate in a BI. Those who consented (*n* = 335) completed an interviewer-administered baseline questionnaire before being randomly assigned to one of three intervention conditions. Participants were assigned to an intervention condition by means of sequentially numbered sealed opaque envelopes, thus ensuring allocation concealment. The randomisation sequence was generated by random number tables by a researcher not involved in the delivery of the intervention. Outcome measures were collected again 3 months after the end of the intervention. One hundred and eighty two of the 335 participants enrolled in the study completed the 3 month assessment. Counsellors who delivered the intervention did not conduct the follow-up assessment and assessors responsible for collecting the outcome measures were blinded to the treatment allocation. A detailed description and graphical depiction of study recruitment, attrition and study methods is provided elsewhere [47].

### Interventions

Participants were assigned to one of three conditions. Participants assigned to the control arm were given a brochure providing information on the effects of substance use. No additional counselling was provided. Participants assigned to the motivational interviewing arm completed a 20-min ASSIST-linked BI developed by the World Health Organisation [49]. Participants assigned to the blended motivational interviewing-problem solving therapy intervention received up to five intervention sessions. During these sessions, the counsellor and the participant collaborated to identify problems occurring in the participant’s life and the counsellor taught the participant a structured approach to addressing these problems. This intervention has been described in detail elsewhere [47, 50].

### Measures

#### Substance use

We used the ASSIST to measure the extent of participant’s substance use involvement which was the primary outcome measure for this study. Scores on the ASSIST can be used to categorise people into low, moderate, or high risk for substance-related health problems. Participants with scores of 0–10 for alcohol and <4 for drugs were categorised as low risk, those with scores of 11–26 for alcohol and 4–26 for drugs were categorised as moderate risk, and those with scores >26 were categorised as high risk for severe substance-related problems [48]. The ASSIST allows for a substance use involvement score to be calculated for each substance of abuse. For this study, our primary outcome measure was changes in the ASSIST score from baseline assessment to 3 month follow-up for the most problematic substance.

We used the baseline findings from the ASSIST to create three dummy variables: alcohol use only, (single) drug use only, or poly-substance use (defined as the use of two or more substances, one of which could be alcohol). We also used the ASSIST to classify participants by type of substance. We created dummy variables for any problem alcohol use, any problem cannabis use, and any problem methamphetamine use. Response options for these variables were coded as yes (1) and no (0).

#### Readiness to change

The Stages of Change, Readiness, and Treatment Eagerness Scale (SOCRATES 8) was used to assess participants’ readiness to change their substance use at baseline. The SOCRATES is a 19-item measure that has been found to have good reliability [51]. It consists of three factorially-derived subscales: Recognition, Ambivalence, and Taking Steps. Recognition scores range from 7–35, with scores from 7 to 31 classified as low and those ≥ 32 as high. Higher scores reflect greater acknowledgment that the individual is having problems related to his or her substance use and that change is needed. Ambivalence scores range from 4 to 20, with scores of 4–14 were classified as low and those ≥ 15 as high. Here, higher scores indicate greater uncertainty about whether or not they want to change. Taking Steps scores range from 8 to 40, with scores of 8–32 classified as low and scores ≥ 33 as high. For this scale, higher scores indicate that the individual has begun making positive changes to his or her substance use.

#### Depression

The Center for Epidemiological Studies Depression Scale (CES-D) was used to measure depressive symptoms at baseline. This tool was designed to measure common symptoms of depression in the general population and consists of 20 self-rated items. Each item is rated on a four-point Likert scale, ranging from 0 (indicating no symptom presence) to 3 (indicating the presence of symptoms most of the time). Composite scale scores range from 0 to 60, with a score of 16 or higher signifying clinically meaningful depression [52].

#### Socio-demographic variables

Information on the socio-demographic characteristics of participants (age, gender, race, marital status, employment status, and level of education) were also collected. Participants who presented with an injury were also asked whether the injury was related to substance use.

### Ethics statement

Ethical approval for this study was provided by the Human Research Ethics Committee from the University of Cape Town's Faculty of Health Science. All participants were required to provide written informed consent to participate in the study prior to study enrolment. The trial on which this study was based was registered with the Pan African Clinical Trial Registry (PACTR201308000591418).

### Analyses

Data were analysed using the Statistical Package for Social Sciences version 22. First we generated descriptive statistics for socio-demographic, substance use, mental health, and RTC variables for the entire sample and the 182 participants who completed the follow-up. Race and intervention condition were the only variables that distinguished between participants who completed the 3 month follow-up and those who did not. Next, for participants who completed the 3 month follow-up (*n* = 182), we conducted simple and multiple linear regression analyses to examine unadjusted and adjusted associations between various baseline socio-demographic, mental health and RTC variables and change in substance use involvement between baseline and 3 month follow-up. Only variables significantly associated with change in ASSIST scores in the simple regression analyses were entered into the multivariable analysis, while controlling for the potential confounding effects of age, gender and race, the intervention condition, and type of substance used. As we were also interested in variables associated with each component of RTC, we used the entire baseline sample (*n* = 335) to examine unadjusted and adjusted associations between various socio-demographic and mental health variables and baseline SOCRATES recognition, ambivalence and taking action scores, respectively. Only variables significantly associated with the dependent variable in the simple regression analyses were entered into the multivariable analysis, while controlling for the potential confounding effects of age, gender and race. The results of the regression models are reported as unstandardized coefficients (*B*) with 95 % confidence intervals (CIs).

## Results

### Characteristics of the sample

Participants were mostly single and male. Black African and Coloured (of mixed race ancestry) participants were equally represented in both samples. Approximately half of the sample had not completed high school and were unemployed (Table 1).

**Table 1:** Baseline socio-demographic, substance use, mental health and readiness to change characteristics of the total (*N* = 335) sample and those who completed the 3 month follow-up (*N* = 182)

	Total Sample	Follow-up sample
	*N* = 335	*N* = 182
Gender (%, n)		
Male	65.5 % (218)	62.1 % (113)
Female	34.5 % (115)	37.9 % (69)
Age (mean, SD)	30.3 (9.6)	30.1 (9.3)
Race (%, n)		
Black	58.9 % (195)	52.0 % (93)
Coloured	40.3 % (135)	48.0 % (86)
Marital Status (%, n)		
Single	82.9 % (272)	84.7 % (150)
Married/attached	17.1 % (56)	15.3 % (27)
Education (%, n)		
Did not finish high school	50.1 % (168)	51.1 % (93)
Finished high school	49.9 % (167)	48.9 % (89)
Employment (%, n)		
Employed	44.6 % (148)	47.5 % (86)
Unemployed	55.4 % (184)	52.5 % (95)
Substance Use Involvement Score^a^ (mean, SD)	19.3 (6.2)	19.6 (6.5)
Problem Alcohol use (%, n)		
Yes (%, n)	86.6 % (290)	86.3 % (157)
No (%, n)	13.4 % (45)	13.7 % (25)
Problem Cannabis use (%, n)		
Yes (%, n)	14.6 % (49)	12.6 % (23)
No (%, n)	85.4 % (286)	87.4 % (159)
Problem methamphetamine use (%, n)		
Yes (%, n)	8.1 % (27)	11.0 % (20)
No (%, n)	91.9 % (308)	89.0 % (162)
Alcohol use only		
Yes (%, n)	78.2 % (262)	77.5 (141)
No (%, n)	21.8 % (73)	22.5 % (41)
Drug use only		
Yes (%, n)	9.0 % (30)	9.3 % (17)
No (%, n)	91.0 % (305)	90.7 % (165)
Poly-substance use		
Yes (%, n)	12.8 % (43)	13.2 % (24)
No (%, n)	87.2 % (292)	86.8 % (158)
Injury related to substance use		
Yes (%, n)	54.2 % (181)	54.1 % (98)
No (%, n)	45.8 % (153)	45.9 % (83)
Depression score (CES-D; mean, SD)	17.6 (10.6)	15.9 (9.9)
SOCRATES Recognition (mean, SD)	19.0 (6.0)	18.7 (6.2)
SOCRATES Ambivalence (mean, SD)	11.2 (3.8)	11.0 (4.0)
SOCRATES Taking Steps (mean, SD)	25.23 (7.2)	25.6 (6.9)

^a^For this variable, the highest scoring substance was used for each participant

Most participants reported problems with alcohol, followed by problems with cannabis and methamphetamine (Table 1). More than two-thirds of participants reported only using alcohol, approximately 13 % reported using multiple substances, and about 9 % reported only using their illicit drug of choice. When risk for substance-related problems was considered, the mean ASSIST score among both the total sample (M = 19.3, SD = 6.2) and among participants who completed the follow-up was within the moderate risk category (M = 19.6, SD = 6.5). Among participants who presented with an injury, about 54 % reported that it was related to their substance use.

When risk for depression was considered, the mean CES-D score was 15.9 (SD = 9.9) among participants who completed the follow-up and 17.6 (SD = 10.6) for the entire sample (Table 1). Readiness to change substance use was low for the majority of the sample. For the total sample and the follow-up sample, the mean scores on the SOCRATES Recognition (M = 19.0, SD = 6.0; M = 18.7, SD = 6.2, respectively), Ambivalence (M = 11.2, SD = 3.8; M = 11.0, SD = 4.1, respectively), and Taking Steps (M = 25.2, SD = 7.2; M = 25.6, SD = 6.9, respectively) scales were low, suggesting that most participants did not recognise that their substance use was problematic, were certain they did not want to change their substance use and were not taking steps to change (Table 1).

### Predictors of change in substance use involvement from baseline to three month follow-up

In unadjusted analyses, the only variables associated with change in substance use involvement from baseline to follow-up were baseline SOCRATES Recognition and Ambivalence scale scores and the intervention condition (Table 2). After adjusting for the effect of the intervention condition, type of substance used, and socio-demographic variables (age, gender, race) that could potentially confound the outcome, the baseline SOCRATES Recognition score remained a significant predictor of change in substance use involvement. Compared to participants with low baseline recognition scores, participants with scores in the high recognition category had on average an 11-point reduction in substance use involvement scores at follow-up (*B* = 11.62; 95 % CI = 6.2–17.0), irrespective of the type of intervention received. In this adjusted model, the SOCRATES Taking Steps score was also a significant predictor of (negative) change in substance use involvement. Compared to participants with low baseline Taking Steps scores, participants with scores in the high category had on average a nine-point increase in substance use involvement at follow-up (*B* = -9.5; 95 % CI = -15.5- -3.5; Table 2), reflecting worsening substance use problems. Alcohol use was the only other significant predictor of change in ASSIST scores (after controlling for the effect of the intervention). Participants with problem alcohol use had, on average, a four-point greater reduction in substance use involvement at follow-up relative to participants who reported other types of substance use problems (*B* = 4.4; 95 % CI = 0.9-7.9; Table 2).

**Table 2:** Unadjusted and Adjusted Linear Regression Models Predicting Change in ASSIST scores from baseline to 3 month follow-up (*n* = 182)

	Unadjusted Analysis			Adjusted Analysis		
Variables	B	95 % CI ^a^	*p*-value	B	95 % CI	*p*-value
Age	0.03	-0.08-0.14	0.56	0.02	-0.09-0.13	0.77
Gender (Male)						
Female	0.98	-1.13-3.08	0.36	1.54	-0.51-3.60	0.14
Race (black)						
Coloured	-0.42	-2.49-1.64	0.69	0.15	-1.96-2.27	0.89
Marital Status (single)						
Married	1.48	-1.38-4.35	0.31			
Education (not complete school)						
Completed high school	0.28	-1.77-2.33	0.79			
Employment						
Unemployed	-1.49	-3.53-0.56	0.15			
Problem Alcohol use (no)						
Yes	1.73	-1.24-4.90	0.25	4.40	0.90-7.90	0.01
Problem Cannabis use (no)						
Yes	-0.001	-3.08-3.08	0.99	1.11	-1.85-4.06	0.46
Problem Methamphetamine (no)						
Yes	1.34	-1.93-4.60	0.42	3.26	-0.89-7.42	0.12
Poly-substance use (no)						
Yes	0.55	-2.5- 3.57	0.72			
Injury related to substance use (no)						
Yes	-0.14	-2.20-1.93	0.90			
Depression score (CES-D)	0.08	-0.02-0.18	0.13			
Readiness to change: Ambivalence (low)						
High	2.18	-0.22-4.57	0.07			
Readiness to change: Recognition (low)						
High	4.55	1.87-7.22	0.001	11.60	6.19-16.19	<0.001
Readiness to change Taking Steps (low)						
High	2.38	-0.68-5.44	0.13	-9.51	-15.52--3.50	0.002
Intervention Condition						
MI vs. Others	-0.97	-3.07-1.13	0.36	1.82	-0.41-4.05	0.11
PST vs. Others	4.28	2.01-6.54	<0.001	5.61	3.07-8.15	<0.001

^a^95 % CI = 95 % confidence interval

### Variables associated with readiness to change

In unadjusted analyses, race, depression, substance use involvement score, having a substance-related injury, cannabis problems, methamphetamine use problems, and poly-substance use were the only variables associated with the baseline SOCRATES Recognition scale scores (Table 3). In the adjusted model, depression remained significantly associated with “Recognition”, with higher CES-D scores associated with greater recognition of the need for change at baseline (*B* = 0.2; 95 % CI = 0.1–0.3). Participants with a substance use injury also had higher levels of problem recognition (*B* = 1.9; 95 % CI = 0.6–3.2). Methamphetamine use was also significantly associated with problem recognition. Participants with methamphetamine problems had on average a 3.4-point higher “Recognition” score (*B* = 3.4; 95 % CI = 0.5–6.3) relative to participants who did not use methamphetamine (Table 3).

**Table 3:** Unadjusted and Adjusted Linear Regression Models Predicting SOCRATES Recognition Scores (*n* = 335)

	Unadjusted Analysis			Adjusted Analysis		
Variables	B	95 % CI^a^	*p*-value	B	95 % CI	*p*-value
Age	0.02	-0.09-0.05	0.57	0.04	-0.02-0.11	0.18
Gender (Male)						
Female	0.60	-0.76-1.97	0.38	1.27	0.02-2.53	0.05
Race (black)						
Coloured	-1.36	-2.66- -0.06	0.04	-0.18	-1.56-1.21	0.80
Marital Status (single)						
Married	-0.73	-2.44-0.98	0.40			
Education (not complete high school)						
Complete high school	-0.2	-1.31-1.27	0.98			
Employment (employed)						
Unemployed	-0.73	-2.03-0.58	0.27			
Substance-related injury						
Yes	1.54	0.26-2.83	0.02	1.87	0.57-3.16	0.005
Depression score (CES-D)	0.23	0.18-0.29	<0.001	0.19	0.12-0.25	<0.001
Substance Use Involvement Score-baseline	0.29	0.20-0.39	<0.001	0.09	-0.02-0.21	0.19
Problem Alcohol use (no)						
Yes	-0.52	-2.44-1.39	0.59			
Problem Cannabis use (no)						
Yes	1.81	0.01-3.61	0.05	0.65	-2.09-3.39	0.64
Problem Methamphetamine (no)						
Yes	4.79	2.45-7.13	<0.001	3.43	0.54-6.32	0.02
Poly-substance use (no)						
Yes	3.46	1.58-5.34	<0.001	2.01	-0.64-4.65	0.14

^a^95 % CI = 95 % confidence interval

Being of Coloured ethnicity, greater depression, greater substance use involvement at baseline, cannabis problems, methamphetamine problems, and poly-substance use were associated with higher baseline SOCRATES Ambivalence scores in unadjusted analyses (Table 4). In the adjusted model, female gender was significantly associated with greater “Ambivalence” (*B* = 1.0; 95 % CI = -0.2-1.9). Participants who self-identified as Coloured had lower scores on the “Ambivalence” scale than those who were Black African (*B* = -1.2; 95 % CI = -2.0- -0.2). Depression also remained significantly associated with ambivalence, with higher CES-D scores associated with greater ambivalence about change (*B* = 0.1; 95 % CI = 0.04-0.1). Methamphetamine use was also significantly associated with ambivalence in this model. On average, participants who reported methamphetamine use had a 2.3-point higher “Ambivalence” scale than participants who did not use methamphetamine (*B* = 2.3; 95 % CI = 0.1-4.2; (Table 4).

**Table 4:** Unadjusted and Adjusted Linear Regression Models Predicting SOCRATES Ambivalence Scores (*n* = 335)

	Unadjusted Analysis			Adjusted Analysis		
Variables	B	95 % CI^a^	*p*-value	B	95 % CI	*p*-value
Age	0.01	-0.05-0.03	0.64	0.03	-0.04-0.08	0.19
Gender (male)						
Female	0.54	-0.33-1.41	0.23	1.04	0.22-1.86	0.01
Race (black)						
Coloured	-0.96	-1.79- -0.13	0.02	-1.16	-1.99--0.24	0.01
Marital Status (single)						
Married	-0.07	-1.75-1.61	0.94			
Education (not completed)						
Completed high school	-0.25	-1.08-0.57	0.55			
Employment						
Unemployed	-0.74	-1.57-0.90	0.08			
substance-related injury						
Yes	0.51	-0.31-1.3390	0.22			
Depression score (CES-D)	0.12	0.08-0.16	<0.001	0.08	0.04-0.12	<0.001
Substance Use Involvement Score Baseline	0.13	0.06-0.19	<0.001	0.03	-0.04-0.10	0.36
Problem Alcohol use (no)						
Yes	-4.63	-1.68-0.76	0.46			
Problem Cannabis use (no)						
Yes	1.42	0.27-2.60	0.02	0.14	-1.66-1.94	0.88
Problem Methamphetamine (no)						
Yes	0.12	0.08-0.16	<0.001	2.34	0.05-4.22	0.02
Poly-substance use						
Yes	2.46	1.25-3.66	<0.001	0.76	0.97-2.48	0.39

^a^95 % CI = 95 % confidence interval

Being of Coloured ethnicity, presenting with an injury that was preceded by substance use, baseline substance use involvement scores, problem cannabis use, problem methamphetamine use and poly-substance use were associated with higher baseline SOCRATES Taking Steps scale scores in unadjusted analyses (Table 5). In the adjusted model, participants who self-identified as Coloured had higher scores on the “Taking Steps” scale than those who were Black African (*B* = 2.2; 95 % CI = 0.6-3.9). In contrast, participants who presented to the ED with an injury that was preceded by substance use scored close to two-points lower on this scale than those who did not present with such an injury (*B* = -1.7; 95 % CI = -5.0-0.6; Table 5). Participants who reported problems with methamphetamine use scored on average four-points higher on this scale relative to those without methamphetamine use (*B* =3.9; 95 % CI = 0.5-7.3). Similarly, participants who reported poly-substance use scored close to six-points higher on this scale relative to those who did not report poly-substance use (*B* =5.7; 95 % CI = 1.6-9.8).

**Table 5:** Unadjusted and Adjusted Linear Regression Models Predicting baseline SOCRATES Taking Steps Scores (*n* = 335)

	Unadjusted Analysis			Adjusted Analysis		
Variables	B	95 % CI^a^	*p*-value	B	95 % CI	*p*-value
Age	0.05	-0.03-0.13	0.24	0.04	-0.04-0.12	0.33
Gender (Male)						
Female	0.32	-1.32-1.96	0.70	0.21	-1.37-1.79	0.78
Race (black)						
Coloured	4.30	2.79-5.80	<0.001	2.24	0.55-3.93	0.01*
Marital Status (Single)						
Married	0.47	-1.60-2.53	0.66			
Education (not completed)						
Completed high school	-0.24	-1.80—1.311.66	0.76			
Employment						
Unemployed	-1.09	-2.66-0.48	0.17			
Substance-related injury						
Yes	-3.65	-5.16 - -2.14	<0.001	-1.66	-4.95- -0.56	0.04
Depression score (CES-D)	-0.06	-0.13- 0.01	0.12			
Substance Use Involvement Score (Baseline)	-0.22	-0.34- -0.09	0.001	-0.16	-0.29-0.02	0.23
Problem Alcohol use (no)						
Yes	-3.82	-6.07- - 1.56	0.001	-3.14	-6.54- 0.25	0.07
Problem Cannabis use (no)						
Yes	2.07	-0.11- 4.25	0.06			
Problem Methamphetamine (no)						
Yes	6.15	3.39-8.92	<0.001	3.88	0.52-7.25	0.02
Poly-substance use						
Yes	3.23	0.94-5.52	0.006	5.69	1.58-9.79	0.007

^a^95 % CI = 95 % confidence interval

## Discussion

To the best of our knowledge, this is the first study to examine how RTC impacts substance use outcomes while controlling for the effect of three different brief interventions delivered in EDs in an African country where infrequent but heavy episodic drinking is typical [2]. Similar to studies conducted in EDs in high-income countries [27–29, 53, 54], this study found that RTC was an important predictor of response to substance use interventions. More specifically, participants who reported greater recognition that their substance use was problematic and of the need for change reported larger reductions in their post-intervention substance use involvement. There are several potential explanations for this association; participants who had more recognition of the need for change at the beginning of the intervention may have paid more attention and been more receptive to the intervention content and intervention materials, may have practiced implementing the skills taught during the intervention more diligently, and may have sought out additional information about how to reduce their substance use involvement than participants with lower levels of recognition. All of these factors may have contributed to participants with higher levels of recognition being more successful at reducing their substance use involvement.

As these findings suggest that people are more likely to be amenable to changing their substance use if they are aware that a problem exists and recognise the need for change, interventions to modify substance use should consider including components that enhance problem recognition and awareness of the need for change. Future adaptations of our interventions may wish to include more use of motivational interviewing techniques, particularly given evidence that the use of motivational interviewing promotes RTC [44, 54]. This seems particularly important for this ED population for whom we found very low levels of problem recognition, suggesting that many participants had not yet considered changing their substance use involvement despite already experiencing moderately severe adverse consequences. These low levels of RTC are not surprising given that participants were seeking health care rather than assistance for substance-related problems. Similar levels of RTC have been observed in other studies of non-treatment-seeking populations [22, 28, 34, 36].

Interestingly, participants with alcohol use problems responded better to the brief substance use interventions than participants with other drug problems. This suggests that although brief interventions may be adequate for helping individuals reduce their alcohol use, they may not be sufficient for helping people change their drug use. This finding is not altogether surprising given evidence from other settings that brief interventions in non-treatment seeking populations are efficacious for reducing alcohol use [13–16], but not for reducing illicit drug use [19–23]. However, it remains unclear why these interventions are effective for reducing alcohol use only. Although not explored here, it is possible that type of substance used may have moderated the effect of problem recognition on substance use outcomes. Future ED studies that are adequately powered should consider testing this hypothesis.

In contrast, participants who had higher baseline scores on the SOCRATES Taking Steps scale (reflecting self-reported action towards changing substance use) reported greater substance use involvement at 3 month follow-up, suggesting that their substance use problems had worsened over time. This finding was surprising as studies with treatment-seeking populations have consistently reported that higher scores on this scale predict greater improvement in substance use outcomes [22, 45, 46]. There are two possible explanations for this finding. First, these earlier studies all measured change in longitudinal substance use outcomes at 18 months to 2 years post-intervention. Changing substance use requires individuals to be motivated to take action and to learn how to overcome barriers to change, which takes time and practice [55]. Consequently, we may not have had a long enough follow-up period to observe a positive association between “Taking Steps” scale scores and substance use involvement. Second, some participants may have already been attempting to reduce their substance use involvement prior to receiving the intervention. It is possible that these participants may have relapsed back to old patterns of substance use during the course of the study, and thus reported greater substance use involvement at follow-up. This second explanation is partially supported by findings that participants with more severe types of substance use problems (specifically poly-substance use or methamphetamine use) scored higher on this component of RTC at baseline, suggesting that they had already made some efforts to change. Therefore the negative association found between action-oriented RTC and substance use outcomes could possibly be accounted for by the fact that people with methamphetamine and other complex types of substance use disorders tend to have poorer responses to substance use interventions than individuals with other types of substance use [25]. Future intervention studies should consider collecting more extensive data on participants’ substance use histories and change attempts to further explore this explanation.

In addition, we explored factors associated with each component of RTC that could potentially serve as targets for future interventions to improve RTC substance use in this population. First, we found that participants presenting with injuries that were related to their substance use had greater recognition of the need for change than those who presented without these injuries. This highlights the value of screening all patients who present with injuries for possible substance-related problems as these injuries may offer a window of opportunity for intervening with patients when they are more likely to recognise the negative consequences of continued substance use. In addition and similar to other studies [22, 36, 55], we found that greater problem severity (as reflected in more symptoms of depression and more complex substance use problems, such as methamphetamine use) was positively associated with more recognition that change was needed. Even though our findings suggest that individuals with greater substance use problem severity may not respond as well to BIs interventions as those with less severe problems, we are not suggesting that these individuals are not provided with a BI. In such instances, it is still useful to intervene given evidence that BIs lead to improved substance use treatment utilisation and lower rates of ED utilisation [24]. Together these findings suggest that a certain level of distress or dissatisfaction with current circumstances is needed before a person will recognise the need to make a change. This is consistent with evidence that depressive symptomatology can sometimes have an adaptive function [56]. Given the key role that mood plays in the recognition of the need for change, interventions to enhance RTC could benefit from not only exploring the potential benefits of changing substance use for health and relationships, but also the potential benefits of change for emotional well-being.

Although some distress may help people acknowledge the need for change, our findings suggest that too much distress may impede action towards change. In this study, more symptoms of depression and complex drug problems (in particular methamphetamine use) were associated with greater ambivalence about whether or not to change substance use involvement. One explanation may lie in findings from previous studies that people with more severe substance use disorders and psychiatric disorders have low self-efficacy for resisting substance use [57]. Lack of confidence in one’s ability to resist substance use has been shown to contribute to ambivalence about change and to negatively influence decisions about whether to reduce substance use [58]. Another explanation may be that people with depression and other mental disorders are more likely to have problem solving deficits and to use substances as a way of avoiding their problems [59]. People who use substances as a coping strategy are more likely to have low self-efficacy for resisting substance use and are less likely to take action towards change [60]. However, as we did not directly assess self-efficacy for resisting substance use or problem solving, these explanations require further evaluation in studies that measure self-efficacy and problem solving as well as RTC. Regardless of the reasons for this finding, there may be some benefits to providing substance-using individuals in psychological distress with additional counselling to help them resolve their ambivalence towards change.

Finally, apart from severity of substance use problems, the only other variable associated with the “Taking Steps” scale was whether participants presented with an injury that was preceded by substance use. Participants who presented with a substance use-related injury had lower scores on this scale than participants who did not present with an injury. The lower levels of action-oriented RTC in this patient population group are not surprising given that they are still experiencing many problems related to their substance use. However, as this is a population at high risk of recurring substance use-related injuries [7], it is important to continue to reach them through interventions that build RTC.

These findings should be considered in the light of some limitations. First, the sample size is relatively small and limited to participants recruited from three EDs in one province; as such findings may not be generalizable to patient populations from other locations. Second, the follow-up period was relatively short and it is not known whether the effects of RTC on substance use involvement persist over time. This is a question for future research. Third, we did not collect detailed information on the type or severity of substance-related injury sustained which may have impacted on our ability to find a significant relationship between injury and change in substance use outcomes. Future studies should consider exploring the relationship between severity of substance-related injury and change in substance use involvement. Fourth, 46 % of enrolled participants failed to provide follow-up data. Attrition analyses suggests that attrition was not associated with RTC or degree of substance use involvement, increasing confidence in our findings. Nonetheless, future studies in ED settings should consider how participant retention can be improved.

## Conclusion

Despite some limitations, findings enhance our understanding of the relationship between RTC and subsequent substance use within the context of a brief intervention for this African population of moderate risk substance users. As such, they may inform the development of interventions for other populations with similar patterns of substance use. Results from this study underscore the importance of RTC for response to substance use interventions. In particular, recognition of problems and the need for change predicts future substance use in this population. Given the normalization of substance use in South Africa, these findings suggest that it may be useful for interventions to include a strong focus on exploring the risks associated with continued substance use and building recognition of the need for change as first steps towards developing commitment to and an action plan for reducing substance use involvement. Findings also suggest that providing additional support to individuals in distress and with severe forms of substance use may enhance intervention outcomes. For participants who are already trying to reduce their substance use, interventions that include relapse prevention strategies may prove effective components of interventions to positively change substance use involvement. Interventions for substance use in this population should assess and address RTC particularly since it is an important construct for predicting successful outcomes, and because it is mutable through motivational enhancement and similar intervention strategies.

### Availability of data and materials

It is our current policy that data from this project and study materials are not publicly shared, but are made available on written request as we are still analysing this dataset. These requests should be directed to Dr Katherine Sorsdahl (katherine.sorsdahl@uct.ac.za).

## Abbreviations

ED: emergency department
BI: brief intervention
LMIC: low-and middle-income country
RTC: readiness to change
